# Local Climate Experts: The Influence of Local TV Weather Information on Climate Change Perceptions

**DOI:** 10.1371/journal.pone.0141526

**Published:** 2015-11-09

**Authors:** Brittany Bloodhart, Edward Maibach, Teresa Myers, Xiaoquan Zhao

**Affiliations:** 1 Department of Psychology, Colorado State University, Fort Collins, Colorado, United States of America; 2 Center for Climate Change Communication, George Mason University, Fairfax, Virginia, United States of America; University of Washington, UNITED STATES

## Abstract

Individuals who identify changes in their local climate are also more likely to report that they have personally experienced global climate change. One way that people may come to recognize that their local climate is changing is through information provided by local TV weather forecasters. Using random digit dialing, 2,000 adult local TV news viewers in Virginia were surveyed to determine whether routine exposure to local TV weather forecasts influences their perceptions of extreme weather in Virginia, and their perceptions about climate change more generally. Results indicate that paying attention to TV weather forecasts is associated with beliefs that extreme weather is becoming more frequent in Virginia, which in turn is associated with stronger beliefs and concerns about climate change. These associations were strongest for individuals who trust their local TV weathercaster as a source of information about climate change, and for those who identify as politically conservative or moderate. The findings add support to the literature suggesting that TV weathercasters can play an important role in educating the public about climate change.

## Introduction

Most Americans perceive climate change to be a distant threat [1–3], but a growing body of research on the human dimensions of climate change indicates that directly experiencing the effects of climate change increases people’s understanding of and engagement in the issue (e.g., [4, 5]). Changes in local weather patterns and seasons are a common way in which people report personally experiencing climate change [4]. Receiving weather and climate information via the local TV weather report may be one way that individuals can easily gain information about climate changes, particularly when they do not spend extended time outdoors. Further, local weather forecasts are an easily accessible venue for science communication, and potentially climate change communication, that is absent of political context, which may help viewers avoid the politicized motivated reasoning that is common in many other climate communication contexts. The purpose of the present research was to examine this possibility.

### Connecting with Climate Change

Global climate change is highly abstract in nature and often difficult to detect at any one moment, as it is measured by average temperature and climate conditions over an extended period of time. As such, it is difficult for individuals to know if they have directly experienced the effects of climate change. Moreover, individuals, particularly those in highly industrialized countries, are often removed from the climate physically, temporally, socially, and by uncertainty [1, 6]. Therefore, climate educators have begun to suggest ways to connect individuals with their own experiences of climate change. Some climate change engagement strategies aim to decrease psychological distance by stressing the likelihood of climate change impacts (e.g., see [7–9]), or connecting individuals with immediate impacts on groups they care about (such as individuals in developing countries, or animals). These strategies show promise; for example, Spence and colleagues [1] found that decreasing psychological distance in general leads to higher concern about climate change, and that decreased social distance in particular leads to a willingness to take action.

Yet another way to connect individuals with climate change is to focus on the local impacts around them. Research has documented that individuals are relatively accurate in perceiving changes in their local climate [10], from changes in average temperature, precipitation, and seasons, to changes in the presence or absence of plants and animals [11, 4, 12]. Farmers in Burkina Faso, for example, have been shown to detect changes in rainfall over as long as a thirty-year period [13]. However, some individuals are less likely than others to experience their own climate, because they do not directly rely on the local climate for their livelihoods or recreation, for example [14–17]. For these individuals, local weather forecasts may present an important opportunity to gain information about changes to the climate.

### Local TV Weather Forecasts as Sources of Information on Climate Change

Local weather information is accessed by individuals for many reasons (e.g., see [18, 19, 20]), and is still most likely to be accessed via local TV news, despite the availability of online information [21, 18, 22]. American adults view local TV weather forecasts, on average, about once a day to gain information such as what type of clothing to wear or for making plans [23]. This can serve as a daily reminder about what the local climate is like, and attentive viewing of local weather forecasts over time may reinforce or extend viewers’ own direct experience of the weather by providing information about systematic climate changes. In addition to changes in temperatures and precipitation, climate change has been associated with an increase in extreme weather events, such as heat waves, floods, and droughts [24]. Abnormal local weather is associated with increased information-seeking about climate change [25, 26], creating an opportunity for TV weather forecasters to help connect individuals with the more extreme impacts of a changing climate.

Local TV weathercasters are well-positioned as climate science educators because they are highly skilled at interpreting and communicating the uncertainty and probability of weather and climate changes. Further, TV weathercasters are trusted by a majority of the public as a source of climate change information [27], who have frequent access to large portions of the public, often at times when people are most open to learning about the connection between weather and climate change [28, 29]. In fact, many weathercasters report feeling as if they are interpreters and educators more than forecasters [30].

### Pairing Local Climate Information with Global Climate Change

Increasing access to information about local climate is important because individuals’ beliefs about global climate change are shaped, in part, by their experience with local weather and climate changes. Many studies have documented the influence of local temperature changes on short-term beliefs in climate change [31–37, 4], and recent, local climate changes can influence awareness of warming trends over time [11]. Individuals often cite local weather when explaining their beliefs about climate change, and changes in beliefs tend to be related to changes in extreme weather [32]. Those who perceive changes in their local climate are more likely to be convinced that climate change is happening [38, 33], be more concerned and perceive greater risks associated with climate change [39, 4], and are more likely to support policies that address climate change [40]. Extreme and unusual weather have precipitated greater pro-environmental attitudes [25], while messages about climate change impacts that emphasize local over distant information can lead to greater climate change engagement [41].

### Motivated Reasoning and Trust as Possible Limitations

A common concern with climate change communication is a backlash or “boomerang” effect among those who are motivated to deny that climate change is an issue. Political ideology and underlying worldviews have repeatedly been shown to influence climate change perceptions in the United States, leading to strong polarization of the issue in this country (e.g., [42–45]). This type of motivated reasoning [43] likely accounts for the findings that those who do not believe in climate change are less likely to perceive changes in their local climate [11] while those who are convinced of climate change are more likely to (accurately) remember local warming trends [46]. Further, those who do not believe in “global warming” are less likely to be concerned about those impacted by climate change, leading to less support for climate mitigation policies [47].

It is therefore possible that climate information communicated through local TV weather forecasts might only influence beliefs about climate change among those in the political middle-ground: independents and those who do not strongly identify with a political party. Past research has indicated that perceived experience of climate change [5] and short-term temperature changes [48] are most likely to influence climate change beliefs among those who did not hold strong beliefs in the first place. However, it is also possible that information about local weather impacts (i.e., information that people can, in part, verify with their own senses) is less susceptible to motivated reasoning than information about distant or global climate changes. Therefore, receiving repeated information about local climate changes may influence beliefs and attitudes of those who are not convinced about the existence of climate change. For example, Goebbert and colleagues [37] found that while actual temperature effects on perceived weather were subject to political motivated reasoning, actual precipitation, which is perhaps less politicized, influenced perceptions of changing weather regardless of political ideology. Recent data supports that climate change beliefs are not entirely socially influenced: controlling for common demographics, political ideology, and beliefs about policy regarding climate change, personal experience with local climate still influences the perceived risk of the global issue of climate change [4].

It is also possible that local weather information will only inform climate change perceptions to the extent that viewers trust TV weathercasters as a source of information on climate change. Messages about climate change tend to be less effective among those who do not trust scientists as reliable sources of information on climate change (e.g., [38, 49]). Instead, research has repeatedly demonstrated that individuals tend to accept a message if the source of the message is perceived to be trustworthy (e.g., [50, 51]). Messages about climate change from elite sources (e.g., TV newscasters, congressional representatives) are strong predictors of climate change attitudes, even in the face of extreme weather events such as the 2010 and 2011 “Snowmageddon” winter events [45, 52]. Despite that TV weathercasters can sometimes be inaccurate in their weather predictions, viewers generally trust their local weathercasters and follow their advice during extreme weather [53]. Specifically, Anderson and colleagues [54] found that viewers were more likely to learn from a short climate education video featuring a local TV weathercaster if they trusted the weathercaster. Therefore, trust in TV weathercasters may play an important role in whether receiving information about local weather affects perceptions about climate change.

### Current Research

**Fig 1:**
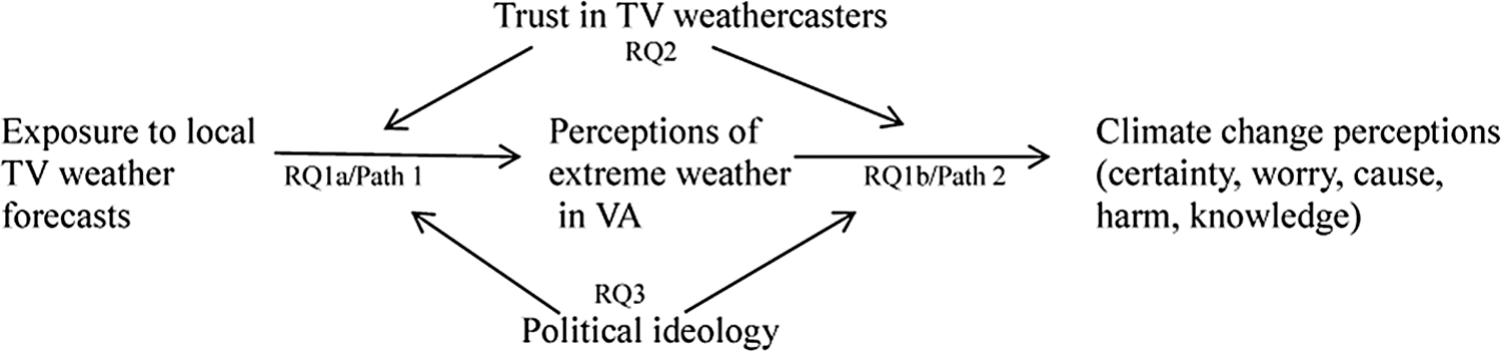
Proposed moderated mediation.

We investigated whether paying attention to local TV weather forecasts directly influences perceptions of extreme local weather, and indirectly influences attitudes and beliefs about climate change. Specifically, we explored the following research questions (see Fig 1):

(a) Does exposure to local TV weather forecasts influence perceptions about changes in the local climate?
  (b) Do perceptions about local climate changes influence more global beliefs about climate change?
Does trust in TV weathercasters moderate the impact of weather information on perceptions about climate change?
Does political ideology moderate the impact of weather information on perceptions about climate change?

## Method

### Participants and Design

Telephone interviews with 2,000 Virginia residents, contacted via random digit dialing, were collected by Princeton Survey Research Associates International (PSRAI) in English and Spanish, with a 12.2% response rate. A total of 16,439 working phone numbers were contacted, of which 2,000 participants agreed to participate in the study. The majority of calls took place on landlines (*n* = 1,800), and a minority on cell phones (*n* = 200). Participants were contacted in one of the seven primary news markets in the state of Virginia: Northern Virginia (suburbs of Washington, D.C.), Lynchburg-Roanoke, Richmond, Charlottesville, Harrisonburg, Norfolk-Portsmouth-Newport News, and the Tri-Cities area. Participants were required to be at least 18 years old, and were asked to give verbal consent over the phone of their willingness to participate in the study. Oral consent was obtained from participants because participants were randomly contacted via phone line and did not participate in person (please see S1 Data for the complete dataset).

Consent was recorded by the interviewer and added to a secure file containing the participant's contact information. This information was kept separately from the file containing all survey responses given by the participant. The current methodology for verbal consent and ethical research with human subjects for this study was approved by George Mason University’s Office for Research Integrity and Insurance [IRB # 530095–2].

#### Demographics

Participants were 54% female and 46% male, and evenly distributed across age groups (age groups were categorized into 10 year increments, with 15–20% of participants falling into each age category). Most participants were Caucasian (67%), while 20% were Black or African American and 6% were Hispanic. Another 6% identified as Asian or Pacific Islander, or indicated another ethnicity. The majority of participants had completed a high school degree (32%) or some college (32%), while 14% had finished college, 14% had completed graduate education, and 8% did not finish high school. Almost half of participants (45%) worked full-time, 13% worked part-time, and 43% were not employed. Finally, 41% of participants politically identified as either somewhat or very conservative, 31% identified as politically moderate, and 29% as somewhat or very liberal. All data was weighted against the same demographics from the 2011 US Census Bureau, and thus should be relatively comparable to the U.S. population in general.

### Procedure

Participants were told that the study was being conducted on behalf of a university located in their state about “some important issues today,” and that it was not a sales call. Participants were asked to verify that they were over 18 years old, which state and county they lived in, and their zip code. They were told that they would be paid $10 to complete the study, which should take around 15 minutes. They were also told that they could earn an additional $20 if they completed a second survey in about six months. Finally, they were asked how often they watched their local TV news. Participants were excluded if they answered “never,” due to the fact that participants were going to be contacted again in future studies on the impact of TV weathercasters sharing climate change information with their local viewers.

During the interview, survey administrators asked participants about their behaviors watching local TV news, their perceptions of the weather in Virginia, their perceptions of climate change, and their trust in sources of information about climate change. Participants were read both the survey questions and possible multiple choice answers, and asked to pick the answer closest to their choice. Administrators could also record answers of “don’t know” and “refused”, although these were not choices given to participants. At the end of the survey, participants were asked about demographic information, how best to be contacted in six months if they wanted to participate again, and thanked for their time.

### Measures

#### Exposure to Local TV Weather Forecasts

Participants were asked: “When you watch local news on TV, how much attention do you pay to the weather forecast? A lot, a moderate amount, a small amount, or none at all.” These responses were coded on a 4-point scale (0 = none at all, 3 = a lot, mean = 2.5, *SD* = .70).

#### Perceptions of Extreme Local Weather

Participants were asked about whether three types of extreme weather were becoming more or less frequent in Virginia: extremely hot days, severe droughts, and intense storms like heavy downpours. Responses choices were “much less frequent,” “somewhat less frequent,” “haven’t noticed any change,” “somewhat more frequent,” and “much more frequent.” Because certain types of extreme weather are more common in certain areas of the state than others, responses from all three types of extreme weather were combined to form an overall “*perceptions of extreme weather*” index which ranged from -6 (responding to all items with “much less frequent”) to +6 (responding to all items with “much more frequent”), mean = 1.52, *SD* = 1.97.

#### Perceptions of Climate Change

Participants were asked if they think climate change is happening (yes, no, don’t know); those who responded affirmatively or negatively were asked how sure they were about their response (extremely sure, very sure, somewhat sure, not at all sure). These items were combined into a 9-point “*certainty*” scale (-4 = extremely sure it isn’t happening, 0 = don’t know, and 4 = extremely sure it is happening, mean = 2.07, *SD* = 2.06).

The *cause* of climate change was assessed by asking participants “Assuming climate change is happening, do you think it is…?” (mostly caused by humans, caused by both humans and changes in the environment, mostly caused by natural changes in the environment, caused by other things, or none of the above because climate change isn’t happening). Hot deck imputation [55] was used for those respondents who answered “don’t know” by randomly assigning responses matched on age and education. Results were calculated both with and without hot deck imputation. Although some effects became slightly stronger or weaker, the directionality and significance of all effects remained the same. The responses were imposed upon a 4-point scale, in which “caused mostly by changes in the environment” and “caused by other things were combined: (0 = “none of the above because climate change isn’t happening, 1 = “caused mostly by changes in the environment/other things”, 2 = “caused by both humans and changes in the environment”, 3 = “caused mostly by humans”, mean = 1.91, *SD* = .93).

Worry was assessed by the question: “How worried are you about climate change: very worried, somewhat worried, not too worried, or not at all worried?” Personal importance was assessed by asking: “How important is the issue of climate change to you personally?” This was measured with a five-point scale from “not at all important” to “extremely important.” An exploratory factor analysis of all outcome measures indicated that these two measures loaded together. They were standardized and then averaged to create a “*worry*” measure (Spearman Brown correlation, *SB*_*K*_ = .72, which ranged from 0–4, mean = 1.72, *SD* = .93.

Participants were also asked whether climate change will harm them personally (a four-point scale from “not at all” to “a great deal”), and when, if ever, they think climate change will harm people in Virginia (“now”, “in 10 years”, “in 25 years”, “in 50 years”, “in 100 years”, or “never”). These two items loaded together so were standardized and averaged to create a “*harm*” measure (*SB*_*K*_ = .63), which ranged from 0–5, mean = 2.15, *SD* = 1.26.

Finally, participants were asked how much they had thought about climate change before today (“a lot”, “some”, “a little”, or “not at all”), and how much they personally would say they know about the issue of climate change (“a lot”, “some”, “not too much”, or “nothing at all”). Again, these two items loaded together, so were standardized and averaged to create a “*knowledge* scale” (*SB*_*K*_ = .65) which ranged from 0–3, mean = 1.77, *SD* = .78.

#### Trust in TV Weathercasters

Participants were asked how much they trust TV weathercasters as a source of information about climate change (using a five-point scale from “strongly distrust,” coded “-2”, to “strongly trust,” coded “2”, with “don’t know” as the midpoint), mean = .61, *SD* = 1.09.

#### Political Ideology

Participants were asked how liberal, moderate, or conservative they considered themselves to be (very conservative, somewhat conservative, moderate, somewhat liberal, or very liberal, coded from -2 to +2), mean = -.17, *SD* = 1.17.

## Results

### Influence of Exposure to Local Weather Information

**Fig 2:**
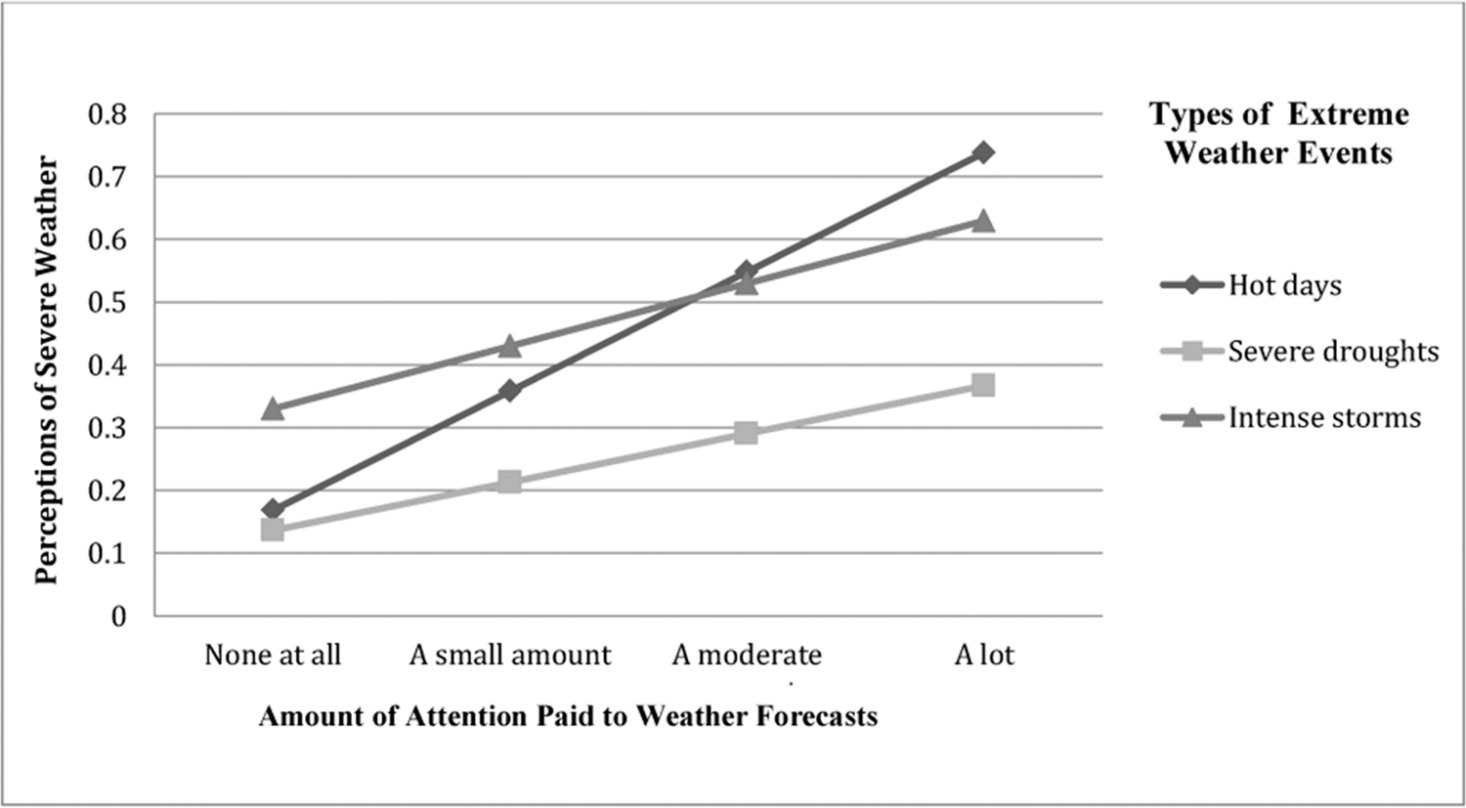
Exposure to local weather information influences perceptions of severe weather in Virginia.

In order to assess RQ1a, perceptions of extreme weather in Virginia were regressed on exposure to local weather information. Those who were exposed to more local TV weather forecasts were more likely to perceive extreme changes in weather than those who paid less attention to weather forecasts. This was true of each extreme weather event (hot days, *B* = .19, *SE* = .03 *p* < .001, *R* ^*2*^ = .02, droughts, *B* = .08, *SE* = .03, *p* < .05, *R* ^*2*^ = .003, severe storms, *B* = .10, *SE* = .03 *p* < .01, *R* ^*2*^ = .01), and extreme weather events overall (index of three types of weather), *B* = .36, *SE* = .06 *p* < .001, *R* ^*2*^ = .02 (see Fig 2). Thus, RQ1a was supported.

**Fig 3:**
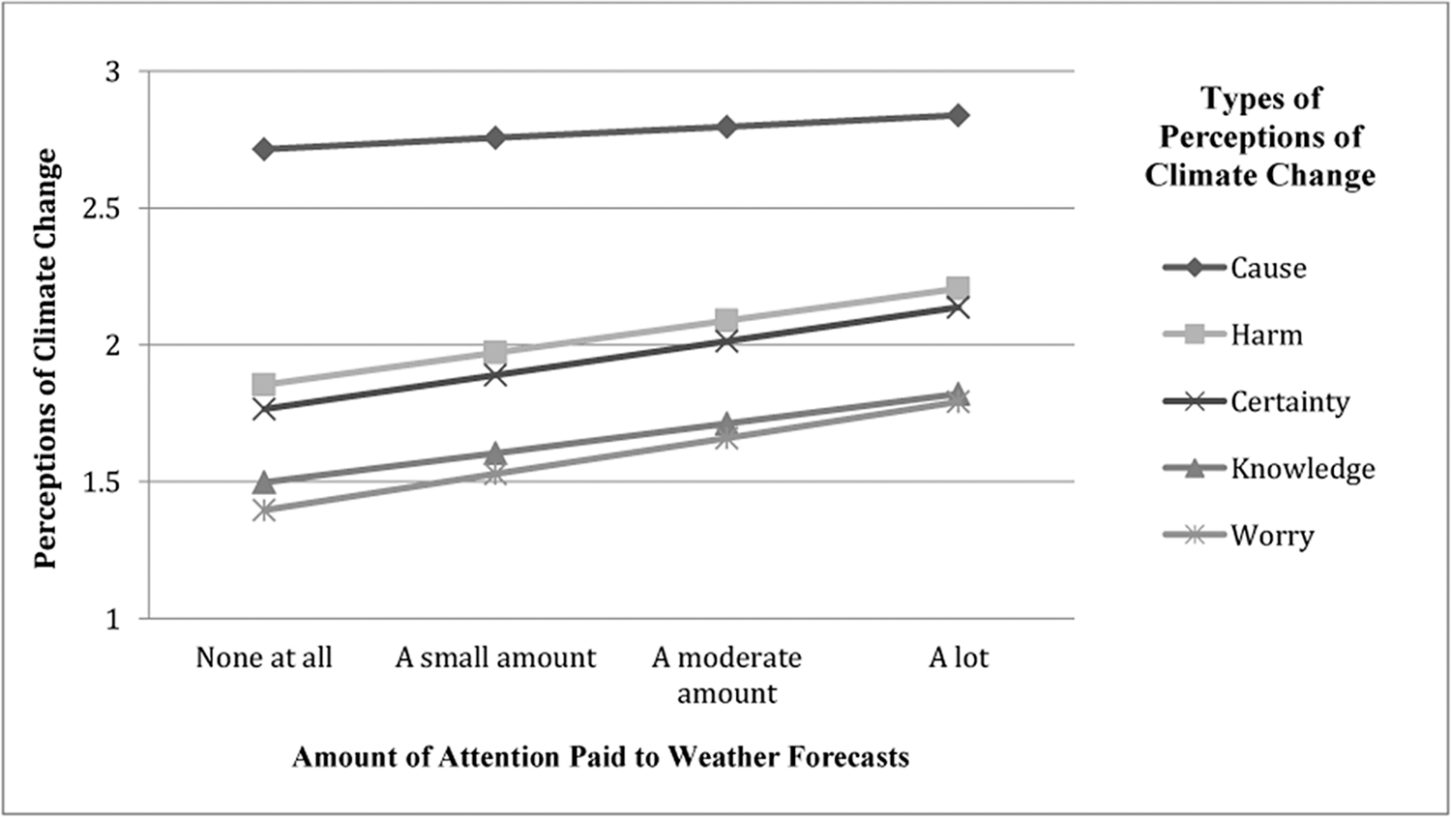
Exposure to local weather information influences perceptions of climate change.

The perceptions of climate change were also regressed on exposure to local weather information, to test for the total influence of exposure to local weather information on climate change beliefs. Results revealed that exposure to weather forecasts predicted worry, *B* = .13, *SE* = .03, *p* < .001, *R* ^*2*^ = .01, harm, *B* = 12, *SE* = .04, *p* < .01, *R* ^*2*^ = .004, and reported knowledge, *B* = .11, *SE* = .03, *p* < .001, *R* ^*2*^ = .01, and marginally predicted certainty that climate change is happening, *B* = .12, *SE* = .07, *p* < .06, *R* ^*2*^ = .002. Exposure did not predict beliefs about the cause of climate change (see Fig 3).

In order to assess RQ1b, we tested whether perceptions of extreme weather in Virginia mediates the effect of exposure to local weather information on the various perceptions of climate change using Hayes’ [56] Mediation Process Model 4. There were positive and significant indirect effects of exposure to local weather information on all perceptions of climate change via perceptions of extreme weather in Virginia: climate change certainty, *indirect effect* = .12, *SE* = .13, *p* < .05; belief about the cause of climate change, *indirect effect* = .05, *SE* = .01, *p* < .05; worry, *indirect effect* = .06, *SE* = .01, *p* < .05; harm, *indirect effect* = .08 *SE* = .02, *p* < .05; and reported knowledge, *indirect effect* = .03, *SE* = .01, *p* < .05. Thus, RQ1b was supported. Further, we tested whether exposure to local weather information directly predicted perceptions of climate change when *controlling* for perceptions of extreme weather (combined index measure). Exposure to local weather information was significantly related to worry, *B* = .07, *SE* = .03, *p* < .05, and knowledge, *B* = .08, *SE* = .01, *p* < .01, but not climate change certainty, beliefs about the cause of climate change, or harm, indicating that perceptions of extreme weather accounted for the association between exposure to weather forecasts and the latter three types of climate change perceptions. For worry and knowledge, the results indicate that exposure to local weather information had some influence on these beliefs independent of perceptions of extreme weather.

Finally, a significant quadratic relationship was observed between local weather information and all five perceptions of climate change measures. Upon probing it was discovered that this quadratic trend was created by those who never watch TV weather forecasts being the most likely to be certain about climate change, worry, feel harmed, and to believe humans are the cause of climate change, and being equally as likely as those who watch weather forecasts “a lot” to report that they are knowledgeable about climate change. However, differences between the “never” and “a lot” groups were not significant, likely due to small a sample size for those who pay no attention to TV forecasts (*n* = 23, out of N = 2,000). We chose to keep a linear prediction because (a) we believe it is plausible that this group is comprised of individuals who receive weather information in other ways (e.g., online) and may not actually be indicative of those who are not exposed to local weather information and (b) the small *n* of those who pay no attention may be overly influencing the shape of the prediction curve. Additionally, a linear pattern is a better test of the theory proposed herein.

### Moderating Effect of Trust in TV Weathercasters

In order to assess RQ2, we tested whether trust in TV weathercasters moderated the indirect effect of exposure to local weather information on perceptions of climate change via perceptions of extreme weather using Hayes’ [56] Conditional Process Macro. Results showed that trust moderated the relationship between exposure to local weather information and perceptions of extreme weather (Path 1), *and* that trust moderated the relationship between perceptions of extreme weather and all five perceptions of climate change measures (Path 2). Specifically, trust and exposure to local weather information interacted to influence perceptions of extreme weather, *B*_Trust*Exposure_ = .12, *SE* = .05, *p* < .05. Next, trust and perceptions of extreme weather interacted to influence climate change certainty, *B*_Trust*EWPerceptions_ = .11, *SE* = .02, *p* < .001, beliefs about the cause of climate change, *B*_Trust*EWPerceptions_ = -.03, *SE* = .01, *p* < .05, worry, *B*_Trust*EWPerceptions_ = -.02, *SE* = .01, *p* < .05, harm, *B*_Trust*EWPerceptions_ = -.05, *SE* = .01, *p* < .001, and reported knowledge, *B*_Trust*EWPerceptions_ = .04, *SE* = .01, *p* < .001. Notably, accounting for trust in TV weathercasters enhanced the effect of exposure to local weather information on extreme weather perceptions, climate change certainty, and knowledge, but somewhat dampened the effect of exposure on the cause of climate change, worry, and harm. The enhancement effects were qualitatively larger in size than the dampening effects.

Next, we probed the interaction to determine the specific indirect effects for those with low trust (-1 standard deviation below the mean), average trust (mean), and high trust (+1 standard deviation above the mean). This demonstrated that for individuals with low trust, there was not a significant indirect effect of exposure to local weather information on any perceptions of climate change, via perceptions of extreme weather. However, there was a significant indirect effect on perceptions of climate change for those with average trust, and even more so for those with high trust, indicating that overall, trust increased the effect of exposure to local weather information on perceptions of climate change (see Table 1). Thus, RQ2 was supported.

**Table 1:** Indirect effects of Exposure to Local Weather Information on Perceptions of Climate Change Measures via Perceptions of Extreme Weather Moderated by Trust in TV Weathercasters.

Outcome Variable	Paths Moderated	-1 SD Trust	Mean Trust	+1 SD Trust
Climate Change Certainty	1 & 2	.054	.095 *	.101 *
Cause of Climate Change	1 & 2	.021	.037 *	.046 *
Worry	1 & 2	.023	.045 *	.062 *
Reported Knowledge	1 & 2	.006	.022 *	.049 *
Harm	1 & 2	.034	.061 *	.076 *

Indirect effects are reported in unstandardized Betas.

* Indicates specific indirect effect significance at *p* < .05.

### Moderating Effect of Political Ideology

In order to assess RQ3, we tested whether political ideology moderated the indirect effect of exposure to local weather information on perceptions of climate change via perceptions of extreme weather using Hayes’ [56] Conditional Process Macro. Moderation of both Paths 1 and 2 were tested conjointly; in cases where the model showed that political ideology only moderated one path, the insignificant interaction was dropped from the model.

Results indicated that political ideology moderated the full proposed mediation model (Paths 1 and 2) for climate change certainty, reported knowledge, and harm, and only the first part of the mediation model (Path 1) for beliefs about the cause of climate change and worry. Specifically, exposure to local weather information and political ideology interacted to influence perceptions of extreme weather, *B*_Ideology*Exposure_ = -.11, *SE* = .05, *p* < .05. Next, perceptions of extreme weather and political ideology interacted to influence climate change certainty, *B*_Ideology*EWPerceptions_ = -.09, *SE* = .02, *p* < .001, harm, *B*_Ideology*EWPerceptions_ = -.04, *SE* = .01, *p* < .001, and reported knowledge, *B*_Ideology*EWPerceptions_ = .05, *SE* = .01, *p* < .001. However, the interaction between perceptions of extreme weather and political ideology was not significant for worry, *B*_Ideology*EWPerceptions_ = -.01, *SE* = .01, *p* = .16, and was marginally significant for beliefs about the cause of climate change, *B*_Ideology*EWPerceptions_ = -.02, *SE* = .01, *p* = .07.

Next, we probed the interaction to determine the specific indirect effects for those who identified as liberal (+1 standard deviation, or a score of “1.0”), moderate/middle of the road (mean, or a score of “-.17”), and conservative (-1 standard deviation, or a score of “-1.34”). In almost all cases, the indirect effect of exposure to local weather information, via perceptions of extreme weather, did not significantly impact perceptions of climate change for liberals, but significantly increased perceptions of climate change for moderates and conservatives, with conservatives having the highest increases in perceptions of climate change among the three groups (see Table 2). One exception is that this indirect effect was not significant for conservatives on the outcome of reported knowledge. Thus, RQ3, which states that political ideology influences the mediated relationship between exposure to local weather information and perceptions of climate change via perceptions of extreme weather, was supported.

**Table 2:** Indirect effects of Exposure to Local Weather Information on Perceptions of Climate Change Measures via Perceptions of Extreme Weather Moderated by Political Ideology.

Outcome Variable	Paths Moderated	Liberal	Moderate	Conservative
Climate Change Certainty	1 & 2	.045	.108 *	.197 *
Cause of Climate Change	1	.029	.054 *	.079 *
Worry	1	.036	.063 *	.090 *
Reported Knowledge	1 & 2	.022	.023 *	.011
Harm	1 & 2	.034	.079 *	.129 *

Indirect effects are reported in unstandardized Betas.

* Indicates specific indirect effect significance at *p* < .05.

## Discussion

Results of the study suggest that exposure to local TV weather forecasts can increase viewers’ perceptions of extreme local weather events, which in turn can increase their awareness about the impacts and reality of climate change. In addition, viewers who have higher trust in TV weathercasters as a source of information about climate change are more likely to be influenced by their exposure to weather forecasts. Although a large number (77%) of participants already trust their local weathercaster as a source of information on climate change, the results highlight the importance of trust in elite sources on perceptions about extreme weather and climate change, and it will be important for climate change communicators, including TV weathercasters, to display and maintain good, scientific credibility.

Further, we found that political ideology influences the strength of exposure to local weather information’s impact on climate change awareness measures, with some surprising findings. Local weather information did not have an apparent influence on liberals, likely due to a ceiling effect. That is, liberals are already likely to report strong beliefs and perceptions about climate change regardless of their exposure to weather information [5]. Conservatives and moderates, on the other hand, *were* affected by exposure to weather information and perceptions of local climate (except for reported knowledge for conservatives, which is in line with previous findings that conservatives report similarly high levels of knowledge about climate change as liberals; [57]). This finding, while encouraging, appears to conflict with previous research on motivated reasoning, which indicates that conservatives do not interpret perceived higher temperatures as a climate pattern or relating to climate change [11]. However, it is possible that the differences in these findings result from differences in the types of weather that participants were asked to recall (e.g., previous research did not ask about “extreme” weather, and found that estimations of precipitation, for example, are less likely to be influenced by beliefs about “global warming” than temperatures), and that hearing about weather from a local weather expert has a different effect on perceptions of weather compared to personal experience alone. Regardless, the current results suggest that exposure to local TV weather forecasts may help to close perceptual gaps about climate change that may otherwise be influenced by political ideology.

One caveat to the above findings was that those who do not pay *any* attention to their local TV weather forecasts (*n* = 23) did not fit the predicted model: instead, this group was just as likely as those who pay a lot of attention to report high levels of perceptions about climate change. It is possible that this group was made up of the small number of individuals who watch their local TV news for other information, but access weather information via other media (e.g., online or through a smart phone). Therefore, their exposure to local weather information may not have been captured by the measure used, and awareness of climate change could be influenced by external sources of information. However, due to the small sample size, we could not statistically explore whether this effect was influenced by age, political ideology, or other demographic characteristics.

### Limitations

The primary limitation to the present research was that people who watch local TV news less than once per week, on average, were excluded from the study. Therefore, our findings pertain only to people who can be considered local TV news viewers, and not the general public. However, the purpose of the research was to understand whether local TV weather forecasts are a viable medium for disseminating climate-based information to the public, and whether this type of information is able to effect subsequent beliefs about climate change on local TV news viewers. Yet it is still important to consider that individuals may receive information about their local weather via other sources (e.g., the internet), and future studies should measure whether this effect continues to occur for alternative forms of exposure to local weather information, especially in light of the current findings supporting the dissemination of information via trusted TV weathercasters.

A second limitation is that participants were not randomly assigned to watch a certain number of weather forecasts, and therefore these results cannot be causally linked to exposure to weather information. Although results support the proposed model, other prediction models may also be plausible (e.g., greater worry about climate change may increase frequency of watching local TV weather forecasts, which may then influence the degree of awareness about extreme weather). While not definitive, results of the interaction of political ideology suggest this is not the case: past research indicates that liberals are more worried about climate change than conservatives [3], yet worry was not related to the frequency of exposure to local weather information for liberals in the current study, but was related to exposure for conservatives.

Third, although we did not ask about the amount of trust participants have for specific weathercasters in their area, we presume that this general measure is likely to capture trust in each participants’ most-watched weathercaster. Prior research has found that the weathercaster is rated the most important person on local TV news broadcasts when choosing which local station to watch [58]. Therefore, our measure of trust in general may be a conservative estimate about trust in comparison to trust in one’s most-watched weathercaster.

### Conclusion

This study adds to the literature which emphasizes the value of education on local climate changes as a way to connect the public with the issue of global climate change, and a small but growing body of research suggesting that TV weathercasters can be an effective source of climate education by virtue of the information they provide. This is especially so given that many members of the public trust TV weathercasters as a source of climate information, and that the effect of the exposure to weather forecasts is moderated by trust in TV weathercasters. That the impact of this coverage is largest on members of society who are least predisposed to know or accept that climate change is occurring (i.e., political conservatives), is an especially important finding because it suggests that TV weathercasters may have an important role to play in bridging the political gap on the issue of climate change. In effect, this may help to move the issue of global climate change out of the realm of the political and back to the realm of the realistic and scientific.

## Supporting Information

S1 Data VA baseline data for publication.sav. Data used for analyses.
(SAV)
